# Preventing surgical site infections: a surgeon's perspective.

**DOI:** 10.3201/eid0702.010214

**Published:** 2001

**Authors:** R L Nichols

**Affiliations:** Tulane University School of Medicine, New Orleans, Louisiana 70112-2699, USA. ronald.nichols@tulane.edu

## Abstract

Wound site infections are a major source of postoperative illness, accounting for approximately a quarter of all nosocomial infections. National studies have defined the patients at highest risk for infection in general and in many specific operative procedures. Advances in risk assessment comparison may involve use of the standardized infection ratio, procedure-specific risk factor collection, and logistic regression models. Adherence to recommendations in the 1999 Centers for Disease Control and Prevention guidelines should reduce the incidence of infection in surgical patients.


### Preventing Surgical Site Infections: A Surgeon's Perspective

**Ronald Lee Nichols**
Tulane University School of Medicine, New Orleans, Louisiana, USA

Wound site infections are a major source of postoperative illness, accounting for approximately a quarter of all nosocomial infections. National studies have defined the patients at highest risk for infection in general and in many specific operative procedures. Advances in risk assessment comparison may involve use of the standardized infection ratio, procedure-specific risk factor collection, and logistic regression models. Adherence to recommendations in the 1999 Centers for Disease Control and Prevention guidelines should reduce the incidence of infection in surgical patients.

Postoperative surgical site infections remain a major source of illness and a less frequent cause of death in the surgical patient (1). These infections number approximately 500,000 per year, among an estimated 27 million surgical procedures (2), and account for approximately one quarter of the estimated 2 million nosocomial infections in the United States each year (3). Infections result in longer hospitalization and higher costs.

The incidence of infection varies from surgeon to surgeon, from hospital to hospital, from one surgical procedure to another, and—most importantly—from one patient to another. During the mid1970s, the average hospital stay doubled, and the cost of hospitalization was correspondingly increased when postoperative infection developed after six common operations (4). These costs and the length of hospital stay are undoubtedly lower today for most surgical procedures that are done on an outpatient basis, such as laparoscopic (minimally invasive) operations or those that require only a short postoperative stay. In these cases, most infections are diagnosed and treated in the outpatient clinic or the patient's home. However, major complications such as deep sternal infections continue to have a grave impact, increasing the duration of hospitalization as much as 20-fold and the cost of hospitalization fivefold (5). Any surgical site infection after open heart surgery results in a substantial net loss of reimbursement to the hospital compared with uninfected cases, a factor that should motivate hospitals to minimize the incidence of postoperative infections (6).

### Description of Surgical Site Infections

The Centers for Disease Control and Prevention (CDC) term for infections associated with surgical procedures was changed from surgical wound infection to surgical site infection in 1992 (7). These infections are classified into incisional, organ, or other organs and spaces manipulated during an operation; incisional infections are further divided into superficial (skin and subcutaneous tissue) and deep (deep soft tissue-muscle and fascia). Detailed criteria for these definitions have been described (7). These definitions should be followed universally for surveillance, prevention, and control of surgical site infections.

Address for correspondence: Ronald Lee Nichols, Tulane University School of Medicine, Department of Surgery SL 22, 1430 Tulane Avenue, New Orleans, LA 70112-2699, USA; fax: 504-586-3843; e-mail: ronald.nichols@tulane.edu

### Microbiology of Surgical Site Infections

The pathogens isolated from infections differ, primarily depending on the type of surgical procedure. In clean surgical procedures, in which the gastrointestinal, gynecologic, and respiratory tracts have not been entered, *Staphylococcus aureus* from the exogenous environment or the patient's skin flora is the usual cause of infection. In other categories of surgical procedures, including clean-contaminated, contaminated, and dirty, the polymicrobial aerobic and anaerobic flora closely resembling the normal endogenous microflora of the surgically resected organ are the most frequently isolated pathogens (8).

According to data from the National Nosocomial Infections Surveillance System (NNIS), there has been little change in the incidence and distribution of the pathogens isolated from infections during the last decade (9). However, more of these pathogens show antimicrobial-drug resistance, especially methicillin-resistant *S. aureus* (10). Postoperative infections, including surgical site infections, were caused by multiple organisms in a multicenter outbreak due to contamination of an intravenous anesthetic, propofol (11). In this outbreak, CDC identified 62 patients at seven hospitals who had postoperative infections, primarily of the bloodstream or surgical site, after exposure to propofol. Only exposure to this anesthetic was substantially associated with these postoperative infections. In six of the seven hospitals, the same pathogen was isolated from several infected patients. The infections were due to extrinsic contamination of the propofol by the anesthesia personnel, who frequently carried the pathogens in lesions on their hands or scalp or in their nares. Lapses in aseptic technique and reuse of single-use vials for several patients were important factors in these outbreaks (11,12). This report stresses the importance of conducting a formal epidemiologic investigation when a cluster of infections involves an unusual organism such as *Moraxella osloensis* or *Serratia marcescens*.

### Prevention of Surgical Site Infections

The most critical factors in the prevention of postoperative infections, although difficult to quantify, are the sound judgment and proper technique of the surgeon and surgical team, as well as the general health and disease state of the patient (13-14). Other factors influence the development of postoperative wound infection, especially in clean surgical procedures, for which the infection rate (<3%) is generally low. Infections in these patients may be due solely to airborne exogenous microorganisms (15).

In 1999, CDC's Health Care Infection Control Practices Advisory Committee published revised guidelines for the prevention of infections (Table 1). This guideline delves extensively into the literature concerning perioperative factors associated with postoperative infections (16). The 1999 edition of the guideline has been extensively revised (Table 2).

### Prophylactic Antibiotic Use in the Surgical Patient

The use of antibiotic prophylaxis before surgery has evolved greatly in the last 20 years (17). Improvements in the timing of initial administration, the appropriate choice of antibiotic agents, and shorter durations of administration have defined more clearly the value of this technique in reducing postoperative wound infections. Some historical milestones of the last 4 decades shed light on the current situation.

### Historical Aspects

Confusing and heated debate concerning the efficacy of prophylactic antibiotics in surgery followed the publication of clinical trials during the 1950s. Errors in study design of these early efforts included nonrandomization, lack of blinding, faulty timing of initial antibiotic administration, prolonged antibiotic use, incorrect choices of antimicrobial agents, and inappropriate choices of control agents.

Experimental studies published during the early 1960s helped clarify many of these problems and resulted in a more scientifically accurate approach to antimicrobial prophylaxis. Most important was the report by Burke (18), which demonstrated the crucial relationship between timing of antibiotic administration and its prophylactic efficacy. His experimental studies showed that to greatly reduce experimental skin infection produced by penicillin-sensitive *S. aureus*, the penicillin had to be in the skin shortly before or at the time of bacterial exposure. This study and others fostered the attitude that to prevent subsequent infection the antibiotic must be in the tissues before or at the time of bacterial contamination. This important change in strategy helped correct the common error of first administering the prophylactic antibiotic in the recovery room.

As early as 1964, Bernard and Cole (19) reported on the successful use of prophylactic antibiotics in a randomized, prospective, placebo-controlled clinical study of abdominal operations on the gastrointestinal tract. The success of antibiotic prophylaxis noted in this early study was clearly due to the authors' appropriate patient selection and wise choice of available agents, as well as the timing of administration. Further advances in understanding of antibiotic prophylaxis in abdominal surgery occurred in the 1970s. During this decade, the qualitative and quantitative nature of the endogenous gastrointestinal flora in health and disease was appropriately defined (20). Many prospective, blinded clinical studies in the 1980s and 1990s prompted

Table 1. Hospital Infection Control Practices Advisory Committee partial recommendations for the prevention of surgical site infection,1999 (16)

| | |
|---|---|
| **Rankings** | |
| Category 1A | Strongly recommended for implementation and supported by well-designed experimental, clinical, or epidemiologic studies |
| Category 1B | Strongly recommended for implementation and supported by some experimental, clinical, or epidemiologic studies and strong theoretical rationale |
| Category II | Suggested for implementation and supported by suggestive clinical or epidemiologic studies or theoretical rationale |
| No recommendation; unresolved issue. | Practices for which insufficient evidence or no consensus regarding efficacy exists |
| **Recommendations—Preoperative—partial and modified** | |
| A. Preparation of the patient | |
| Category 1A | Treat remote infection before elective operation; postpone surgery until treated; Do not remove hair from operative site unless necessary to facilitate surgery; If hair is removed, do immediately before surgery, preferably with electric clippers |
| Category 1B | Control serum blood glucose perioperatively; Cessation of tobacco use 30 days before surgery; Do not withhold necessary blood products to prevent SSIs; Shower or bath on night before operative procedure; Wash incision site before performing antiseptic skin preparation with approved agent |
| Category II | Prepare skin in concentric circles from incision site; Keep preoperative stay in hospital as short as possible |
| Unresolved | Improve nutritional status; Use of mupirocin in nares; Improve oxygenation of wound space; Taper or discontinue systemic steroid use before elective surgery |
| B. Antimicrobial prophylaxis | |
| Category 1A | Select (if indicated) an antimicrobial agent with efficacy against expected pathogen; Intravenous route used to ascertain adequate serum levels during operation and for at most a few hours after incision closed; Before elective colorectal operations, in addition to parenteral agent, mechanically prepare the colon by use of enemas and cathartics. Administer nonabsorbable oral antimicrobial agents in divided doses on the day before the operation |
| Category 1B | Do not routinely use vancomycin for antimicrobial prophylaxis |

SSI = surgical site infections

Table 2. Changes in CDC surgical site infections prevention guidelines, 1999 (16)

| 1985 | 1999 |
|---|---|
| Category 1 | Category 1A |
| Category II | Category 1B |
| Category III | Category II or no recommendation; unresolved |
| **Preoperative hair removal** | |
| Do not remove hair unless it will interfere with the operation<br>Category II | Recommendation unchanged<br>Category 1A |
| If removed, remove by clipping or use of a depilatory, not by shaving<br>Category II | If removed, preferably remove immediately before the operation with electric clippers<br>Category 1A |
| **Preoperative shower or bath** | |
| Patient should bathe with antimicrobial soap the night before an elective operation<br>Category III | Require patients to shower or bathe with an antiseptic agent at least the night before surgery<br>Category 1B |
| **Preoperative hand and forearm antisepsis** | |
| Perform surgical scrub for at least 5 minutes before first operation of day<br>Category 1 | Perform surgical scrub for at least 2-5 minutes with an appropriate antiseptic<br>Category 1B |
| Between consecutive operations perform surgical scrub 2 to 5 minutes<br>Category II | |
| After scrub, dry hands with sterile towel, don sterile gown and gloves<br>Category 1 | After scrub, keep hands up and away from body; dry hands with sterile towel; don sterile gown and gloves<br>Category 1B |
| **Preoperative patient preparation** | |
| Treat and control all bacterial infections before operation<br>Category 1 | Identify and treat all remote infections before elective operation<br>Category 1A |
| The hospital stay should be as short as possible<br>Category II | Keep hospital stay as short as possible<br>Category II |
| If patient is malnourished, enteral or parenteral nutrition should be given<br>Category II | No recommendation to use nutritional support solely to prevent surgical site infection<br>Unresolved |
| **Preoperative antimicrobial prophylaxis** | |
| Use for operations with high infection rate or for those with severe or life-threatening consequences if infection occurs<br>Category 1 | Administer antimicrobial agent only when indicated and select based on published recommendations for a specific operation and efficacy against most common pathogens<br>Category 1A |
| Select antimicrobial agents that are safe and effective<br>Category 1 | |
| Start parenteral IV antimicrobial agents shortly before operation and discontinue shortly afterward<br>Category 1 | Administer antimicrobial agents by IV timed to ensure bactericidal serum and tissue levels when incision made<br>Category 1A |
| | Maintain therapeutic levels during operation and, at most, a few hours after closure<br>Category 1A |
| | Before colorectal elective operations, in addition to IV antimicrobial drugs, mechanically prepare the colon with enemas and cathartic agents; administer nonabsorbable oral antimicrobial agents in individual doses the day before surgery<br>Category 1A |
| | For cesarean sections in patients at high risk administer IV antimicrobial agent immediately after cord is clamped<br>Category 1A |
| | Do not routinely use vancomycin for prophylaxis<br>Category 1B |

definitive recommendations concerning the proper approaches to antibiotic prophylaxis in surgery (21).

### Current Use of Parenteral Antibiotic Agents in Surgical Prophylaxis

The choice of parenteral prophylactic antibiotic agents and the timing and route of administration have become standardized on the basis of well-planned prospective clinical studies (21). It is generally recommended in elective clean surgical procedures using a foreign body and in clean-contaminated procedures that a single dose of cephalosporin, such as cefazolin, be administered intravenously by anesthesia personnel in the operative suite just before incision. Additional doses are generally recommended only when the operation lasts longer than 2 to 3 hours. Other controversial areas include the routine use of antibiotic prophylaxis in clean surgical procedures, such as hernia repair or breast surgery (21,22). This subject has been summarized in a published review (23), and some specific situations will be described.

### Antibiotic Prophylaxis before Elective Colon Resection

The human colon and distal small intestine contain an enormous reservoir of facultative and anaerobic bacteria, separated from the rest of the body by the mucous membrane. A reliable method of sterilizing the colonic contents has been a goal of surgeons throughout this century (24). In the past 25 years, clinical trials have demonstrated that to substantially reduce septic complications after elective colon surgery, antibiotics must have activity against both colonic aerobes (e.g., *Escherichia coli*) and anaerobes (e.g., *Bacteroides fragilis*), a finding we reported over 25 years ago (25). Today, approaches to mechanical cleansing differ widely (26). Modern approaches include standard outpatient mechanical cleansing with dietary restriction, cathartics, and enemas for a 2-day period, or whole-gut lavage with an electrolyte solution of 10% mannitol, Fleet's phospho-soda, or polyethylene glycol, done the day before the operation.

Most surgeons use both antibiotics and mechanical cleansing for preoperative preparation before elective colon resection (26). Three regimens of oral agents combine neomycin with erythromycin base, metronidazole, or tetracycline. The most popular regimen in the United States has been the neomycin-erythromycin base preparation, which was introduced in 1972 (27).

In a survey published in 1997, 471 (58%) of 808 board-certified colorectal surgeons described their bowel preparation practices before elective procedures (26). All respondents used mechanical preparation: oral polyethylene glycol solution (70.9% of respondents), oral sodium phosphate solution with or without bisacodyl (28.4%), and accepted methods of dietary restriction, cathartics, and enemas (28.4%). Most (86.5%) surgeons added both oral and parenteral antibiotics to the regimen; 11.5% added only parenteral antibiotics, 1.1% added only oral antibiotics, and 0.9% did not add antibiotics. Oral neomycin and erythromycin or metronidazole were combined with a perioperative parenteral antibiotic by 77.8% of respondents. Most patients started the preparation as outpatients the day before surgery, and parenteral drugs were added to the regimen 1 to 2 hours before the procedure. The use of outpatient bowel preparation is increasing; however, patient selection is critical, and education is needed to reduce the rate of complications.

### Antibiotic Prophylaxis for Appendectomy

The pathologic state of the appendix is the most important determinant of postoperative infection (28,29). Wound infection after appendectomy for perforative or gangrenous appendicitis is four to five times higher than for early disease. A prospective study of nonperforated appendicitis, using a logistic regression analysis of risk factors, showed that the risk for postoperative infection is related to lack of perioperative antibiotic prophylaxis and to the determination that the appendix was gangrenous (29). Because the pathologic state of the appendix often cannot be determined before or during operation, a parenteral antibiotic agent is recommended as prophylaxis in all patients.

Regimens with activity against both facultative gram-negative bacilli and anaerobes are more effective than those active only against aerobes (29). The use of antimicrobial agents in perforated appendicitis with evidence of local or general peritonitis or intraabdominal abscess, or both, should be considered therapeutic rather than prophylactic.

### Preventive Antibiotics in Penetrating Abdominal Trauma

Hollow-lumen visceral damage with associated escape of endogenous microorganisms is the main risk factor for postoperative infections after exploratory laparotomy for penetrating abdominal trauma. A single dose of parenterally administered antibiotic, given just before abdominal exploration for penetrating abdominal trauma, is associated with low postoperative infection rate in patients with no observed gastrointestinal leakage (30). If gastrointestinal leakage is identified at the time of the operation, continuing the antibiotic agents for 1 to 3 days is usually recommended. It is important to use antibiotic agents with both facultative and anaerobic activity. Leaving the operative wound open, packed with saline-soaked gauze, decreases the incidence of postoperative wound infection in patients at high risk (31).

### Preventive Antibiotic Use in Traumatic Chest Injuries

Recently published studies have shown the value of parenteral antibiotic prophylaxis in the prevention of pneumonia or empyema after the placement of a chest tube to correct the hemopneumothorax associated with chest trauma (32,33). In one study, 500 mg of cefazolin was given intravenously every 8 hours for 24 hours (32). In the other study, 1 g of cefonicid was administered every 24 hours until the chest tube was removed, usually before 5 days (33). In both studies patients receiving antibiotics had substantially lower infection rates than those receiving placebos.

### Conclusions

Recent improvements in antibiotic prophylaxis, including the timing of initial administration, appropriate choice of antibiotic agents, and shortening the duration of administration, have established the value of this technique in many clinical surgical settings. Future study designs should strongly consider risk factors for individual patients when new antibiotic agents are tested or administration techniques are refined. A concentrated effort should be made in areas of clinical surgery where the value of antibiotic prophylaxis has not been proven. A single-dose systemic regimen of an appropriately chosen cephalosporin given during the immediate preoperative period is safe and the indicated practice.

Dr. Nichols is William Henderson Professor of Surgery and Professor of Microbiology and Immunology at Tulane University School of Medicine. He is president of the National Foundation for Infectious Diseases and a past member of the CDC Hospital Infection Control Practices Advisory Committee.

### References

1. Nichols RL. Postoperative infections in the age of drug-resistant gram-positive bacteria. Am J Med 1998;104:11S-16S.
2. Centers for Disease Control and Prevention, National Center for Health Statistics Vital and Health Statistics, Detailed diagnoses and procedures national hospital discharge survey 1994. Vol 127. Hyattsville (MD): Department of Health and Human Services; 1997.
3. Haley RW, Culver DH, White JW, Morgan WM, Emori TG. The nationwide nosocomial infection rate: a new need for vital statistics. Am J Epidemiol 1985;121:159-67.
4. Green JW, Wenzel RP. Postoperative wound infection: a controlled study of the increased duration of hospital stay and direct cost of hospitalization. Ann Surg 1977;185:264-8.
5. Taylor GJ, Mikell FL, Moses HW, Dove JT, Katholi RE, Malik SA. Determinants of hospital charges for coronary artery bypass surgery: the economic consequences of postoperative complications. Am J Cardiol 1990;65:309-13.
6. Boyce JM, Potter-Bynoe G, Dziobek L. Hospital reimbursement patterns among patients with surgical wound infection following open heart surgery. Infect Control Hosp Epidemiol 1990;11:89-93.
7. Horan TC, Gaynes RP, Martone WJ, Jarvis WR, Emori TG. CDC definitions of nosocomial surgical site infections 1992: a modification of CDC definitions of surgical wound infections. Infect Control Hosp Epidemiol 1992;13:606-8.
8. Nichols RL. Prevention of infection in high risk gastrointestinal surgery. Am J Med 1984;76:111-9.
9. Centers for Disease Control and Prevention. National Nosocomial Infections Surveillance (NNIS) report, data summary from October 1986-April 1996, issued May 1996. A report from the National Nosocomial Infections Surveillance (NNIS) System. Am J Infect Control 1996;24:380-8.
10. Schaberg DR. Resistant gram-positive organisms. Ann Emerg Med 1994;24:462-4.
11. Bennett SN, McNeil MM, Bland LA, Arduino MJ, Villarino ME, Perrotta DM. Postoperative infections traced to contamination of an intravenous anesthetic, propofol. N Engl J Med 1995;333:147-54.
12. Nichols RL, Smith JW. Bacterial contamination of an anesthetic agent. N Engl J Med 1995;333:184-5.
13. Nichols RL. Postoperative wound infection. N Engl J Med 1982;307:1701-2.
14. Nichols RL. Surgical wound infection. Am J Med 1991;91 Suppl 3B:54S-64.
15. Nichols RL. Techniques known to prevent postoperative wound infection. Infect Control 1982;3:34-7.
16. Mangram AJ, Horan TC, Pearson ML, Silver LC, Jarvis WR, the Hospital Infection Control Practices Advisory Committee. Guideline for prevention of surgical site infection 1999. Infect Control Hosp Epidemiol 1999;20:247-80.
17. Nichols RL. Surgical infections: prevention and treatment—1965 to 1995. Am J Surg 1996;172:68-74.
18. Burke JF. The effective period of preventive antibiotic action in experimental incision and dermal lesions. Surgery 1961;50:161-8.
19. Bernard HR, Cole WR. The prophylaxis of surgical infection: the effect of prophylactic antimicrobial drugs on the incidence of infection following potentially contaminated operations. Surgery 1964;56:151-9.
20. Nichols RL. Surgical bacteriology: an overview. In: Nyhus LM, editor. Surgery annual. Vol 13. New York: Appleton-Century-Crofts; 1981. p. 205-38.
21. Antimicrobial prophylaxis in surgery. Med Lett Drugs Ther 1999;41:75-80.
22. Platt R, Zalenik DF, Hopkins CC, Dellinger EP, Karchmer AW, Bryan CS. Perioperative antibiotic prophylaxis for herniorrhaphy and breast surgery. N Engl J Med 1990;322:153-60.
23. Nichols RL. Antibiotic prophylaxis in surgery. Current Opinion in Infectious Diseases 1994;7:647-52.
24. Nichols RL, Condon RE. Preoperative preparation of the colon. Surg Gynecol Obstet 1971;132:323-37.
25. Nichols RL, Condon RE. Antibiotic preparation in the colon: failure of commonly used regimens. Surg Clin North Am 1971;51:223-31.
26. Nichols RL, Smith JW, Garcia RV, Waterman RS, Holmes JWC. Current practices of preoperative bowel preparation among North American colorectal surgeons. Clin Infect Dis 1997;24:609-19.
27. Nichols RL, Condon RE, Gorbach ST, Nyhus LM. Efficacy of preoperative antimicrobial preparation of the bowel. Ann Surg 1972;176:227-32.
28. Bennion RS, Thompson JE, Baron EJ, Finegold SM. Gangrenous and perforated appendicitis with peritonitis: treatment and bacteriology. Clin Ther 1990;12 Suppl C:31-44.
29. Browder W, Smith JW, Vivoda L, Nichols RL. Nonperforative appendicitis: a continuing surgical dilemma. J Infect Dis 1989;159:1088-94.
30. Nichols RL, Smith JW, Klein DB, Trunkey DD, Cooper RH, Adinolfi MF. Risk of infection after penetrating abdominal trauma. N Engl J Med 1984;311:1065-70.
31. Nichols RL, Smith JW, Robertson GD, Muzik AC, Pearce P, Ozmen V. Prospective alterations in therapy for penetrating abdominal trauma. Arch Surg 1993;128:55-64.
32. Cant PJ, Smyth S, Smart DO. Antibiotic prophylaxis is indicated for chest stab wound requiring closed tube thoracotomy. Br J Surg 1993;80:464-6.
33. Nichols RL, Smith JW, Muzik AC, Love EJ, McSwain NE, Timberlake G. Preventive antibiotic usage in traumatic thoracic injuries requiring closed tube thoracotomy. Chest 1994;106:1493-8.
